# ILB^®^ Attenuates Clinical Symptoms and Serum Biomarkers of Oxidative/Nitrosative Stress and Mitochondrial Dysfunction in Patients with Amyotrophic Lateral Sclerosis

**DOI:** 10.3390/jpm11080794

**Published:** 2021-08-14

**Authors:** Giacomo Lazzarino, Renata Mangione, Antonio Belli, Valentina Di Pietro, Zsuzsanna Nagy, Nicholas M. Barnes, Lars Bruce, Bernardo M. Ropero, Lennart I. Persson, Benedetta Manca, Miriam Wissam Saab, Angela M. Amorini, Barbara Tavazzi, Giuseppe Lazzarino, Ann Logan

**Affiliations:** 1 UniCamillus, Saint Camillus International University of Health Sciences, 00131 Rome, Italy; giacomo.lazzarino@unicamillus.org; 2 Department of Basic Biotechnological Sciences, Intensive and Perioperative Clinics, Catholic University of Rome, 00168 Rome, Italy; renata.mangione@unicatt.it; 3 Fondazione Policlinico Universitario A. Gemelli IRCCS, 00168 Rome, Italy; 4 College of Medical and Dental Sciences, University of Birmingham, Birmingham B15 2TT, UK; a.belli@bham.ac.uk (A.B.); v.dipietro@bham.ac.uk (V.D.P.); z.nagy@bham.ac.uk (Z.N.); n.m.barnes@bham.ac.uk (N.M.B.); 5 Tikomed AB, 263 03 Viken, Sweden; lars.bruce@tikomed.com; 6 Department of Clinical Neuroscience, Institute of Neuroscience and Physiology, The Sahlgrenska Academy, University of Gothenburg, 413 90 Gothenburg, Sweden; bernardo.mitre@vgregion.se (B.M.R.); lennartpersson@msn.com (L.I.P.); 7 Department of Pharmacy and Biotechnology (FaBiT), University of Bologna, 40126 Bologna, Italy; benedetta.manca4@unibo.it; 8 Department of Biomedical and Biotechnological Sciences, Division of Medical Biochemistry, University of Catania, 95123 Catania, Italy; mirisaab@gmail.com (M.W.S.); amorini@unict.it (A.M.A.); 9 Biomedical Sciences, Warwick Medical School, University of Warwick, Coventry CV4 7AL, UK; 10 Axolotl Consulting Ltd., Droitwich WR9 0JS, UK

**Keywords:** amyotrophic lateral sclerosis, low molecular weight-dextran sulphate, serum biomarkers, energy metabolism, mitochondrial dysfunction, N-acetylaspartate, amino acids, oxidative/nitrosative stress, antioxidants, HPLC

## Abstract

Oxidative/nitrosative stress and mitochondrial dysfunction is a hallmark of amyotrophic lateral sclerosis (ALS), an invariably fatal progressive neurodegenerative disease. Here, as an exploratory arm of a phase II clinical trial (EudraCT Number 2017-005065-47), we used high performance liquid chromatography(HPLC) to investigate changes in the metabolic profiles of serum from ALS patients treated weekly for 4 weeks with a repeated sub-cutaneous dose of 1 mg/kg of a proprietary low molecular weight dextran sulphate, called ILB^®^. A significant normalization of the serum levels of several key metabolites was observed over the treatment period, including N-acetylaspartate (NAA), oxypurines, biomarkers of oxidative/nitrosative stress and antioxidants. An improved serum metabolic profile was accompanied by significant amelioration of the patients’ clinical conditions, indicating a response to ILB^®^ treatment that appears to be mediated by improvement of tissue bioenergetics, decrease of oxidative/nitrosative stress and attenuation of (neuro)inflammatory processes.

## 1. Introduction

Amyotrophic lateral sclerosis (ALS, also known as Lou Gehrig’s disease) is the most common type of motor neuron disease. It is invariably a fatal disease, affecting most populations of motor neurons. The most important degeneration of neuronal cells occurs in motor neurons in the spinal cord, brain stem and brain. The disease begins focally in the central nervous system and then spreads relentlessly [1]. The clinical diagnosis, defined by progressive signs and symptoms of upper and lower motor neuron dysfunction, is confirmed by clinical findings, electromyography, blood and cerebrospinal fluid (CSF) analysis. Although the disease is heterogeneous, most patients die of respiratory muscle weakness less than 3–5 years from symptom-onset. Like other age-related neurodegenerative diseases, ALS has genetic, metabolic and environmental triggers.

As yet, there is no cure for ALS, and management is focused on a combination of neuroprotective medication, multidisciplinary clinics and respiratory support. To date, there is one medication (riluzole (Rilutek^®^), approved originally in the USA in 1995) with anti-glutamatergic properties that prolongs survival, although the effect is limited typically to a few months of additional survival in ALS [2]. Numerous trials have so far been unable to identify any agent that reverses or even halts symptoms. Researchers now aim to slow disease progression by targeting known pathophysiological pathways or genetic defects.

The deranged neuronal function that is associated with the oxidative/nitrosative stress and mitochondrial dysfunction that characterizes the pathophysiological progress of neurodegenerative conditions such as ALS [3] is reflected by changes in related metabolites in blood. When measured, these metabolites can be used as biomarkers of tissue function and, therefore, of disease progression and/or patient response to treatment [4].

The proprietary low molecular weight dextran sulphate (LMW-DS) under investigation in this study is a novel patented formulation of a modified glycosaminoglycan called ILB^®^ (Tikomed AB, Viken, Sweden), which exerts neurotrophic effects through the release and modulation of growth factors including HGF [5] and has been shown to restore brain energy metabolism in the injured brain after severe traumatic brain injury in rats [6].

Here, we report that repeated ILB^®^ administration over four weeks leads to a significant attenuation of the levels of key serum metabolites related to neural damage, oxidative/nitrosative stress and mitochondrial derangement in a cohort of patients with ALS who had participated in the clinical trial entitled ‘A single-center, open single-arm study on the safety, tolerability and efficacy of subcutaneously administered ILB^®^ in patients with amyotrophic lateral sclerosis’; trial registration: EudraCT Number 2017-005065-47. The primary outcomes of this clinical trial are reported elsewhere [7].

## 2. Materials and Methods

### 2.1. Trial Oversight

The clinical trial was a phase IIa, single-center, open label, single-arm proof of concept study of 13 heterogeneous patients with ALS of intermediate disease severity, with safety and tolerability of subcutaneously (s.c.) administered ILB^®^ as the primary endpoints. The study (EudraCT number 2017-005065-47) was conducted at the Sahlgrenska University Hospital, Gothenburg, Sweden. The trial was overseen and approved by the Ethics Committee of the University of Gothenburg and by the Swedish Medical Products Agency (reference number 21,788). The trial was sponsored by Tikomed AB, who had no influence on the conduct of the trial and was not involved in data collection or analysis. The study protocol is described in Supplementary Table S1, and the verbal and written information provided to the patients were in accordance with the Declaration of Helsinki. The underpinning data that support the findings in this study are available from the EU Clinical Trials Register [8].

### 2.2. Patients and Controls

Thirteen patients of either sex with a definite diagnosis of sporadic or genetic forms of ALS and either slow or rapid progression were recruited into the ILB^®^ clinical trial at the Sahlgrenska University Hospital. The male:female ratio was 10:3, mean age ± SD was 56.5 ± 13.3 years and mean ALSFRS-R score ± SD at screening was 36.3 ± 6.7. Individuals were included in the drug trial after giving informed written consent after the diagnosis of ALS was confirmed as definite according to the El Escorial criteria, if there was no other major degenerative or inflammatory disease and if there was a ventilatory capacity of no less than 65% of normal predicted Forced Vital Capacity (FVC) at screening. Patients had to be free of riluzole or lamotrogine for a minimum of 28 days for inclusion in the trial. Full details of the inclusion/exclusion criteria are described in Supplementary Table S1 and elsewhere [8].

A group of 163 age and sex matched healthy subjects (58 ± 14 years, 111 males and 52 females), recruited during the last three years at the Catholic University of Rome among the personnel who underwent the annual health check-up, were used as controls. Written informed consent was obtained from each participant according to the Declaration of Helsinki.

### 2.3. Clinical Assessment of Patients with ALS Using the ALSFRS-R

Disease progression during the clinical trial was assessed at each visit using ALSFRS-R scores, a physician-generated validated assessment of the patient’s degree of functional impairment, which was evaluated serially to assess objectively any impact of treatment on the progression of disease [9]. The ALSFRS-R included questions and observations that allowed the patient’s level of functional impairment in performing 12 aspects of physical function, including speech, salivation, swallowing, handwriting, cutting food, climbing stairs, turning in bed, walking, dressing and hygiene, difficulty in breathing, shortness of breath while lying down and breathing insufficiency. Tasks were rated on a five-point scale from 0 = cannot do, to 4 = normal ability. Individual item scores were summed to produce a reported score of between 0 = worst and 48 = best.

### 2.4. Investigational Medicinal Product (IMP), Dosing and Administration

The active pharmaceutical ingredient of the IMP was an LMW-DS having a mean molecular weight of 5 kDa containing molecules spanning approximately 3–8 kDa with on average 20% sulphation. ILB^®^ is a unique and distinct LMW-DS formulation whose structure, formulation, synthesis and structure has been previously described in a published patent document (publication number: WO 2016/076780—New dextran sulphate). ILB^®^ was provided by Tikomed AB in 10 mL vials containing a solution of 20 mg/mL ILB^®^ in 9 mg/mL NaCl. A single batch of drug was used throughout the study (batch #8059701). ILB^®^ was injected s.c. on alternating sides of the abdomen by the clinical personnel at the Sahlgrenska University Hospital. Five injections of 1 mg/kg, with a weekly dosing interval, were administered in total over 29 days. The exact dose administered depended on the patient’s body weight immediately prior to the first ILB^®^ administration.

### 2.5. Serum Sampling

Blood samples were taken for metabolic biomarker analysis immediately prior to the first ILB^®^ injection at day 0 (pre-treatment: Pre) and again at day 36 (one week post-treatment: Post). Peripheral venous blood samples were collected from both patients and controls after at least 15 min of complete rest, using the standard tourniquet procedure, from the antecubital vein into a single VACUETTE^®^ polypropylene tube containing serum separator and clot activator (Greiner-Bio One GmbH, Kremsmunster, Austria). After 30 min at room temperature, blood withdrawals were centrifuged at 1890× *g* for 10 min and the resulting serum samples saved at temperatures not higher than −20 °C until analysis.

### 2.6. List of ALS Patient Samples, Sample Processing and Analyses

The laboratory received, processed and analyzed serum samples from the patients with ALS (coded as reported in Table 1) in blind. A progressive number, from 1 to 26 was given to each sample. After all analyses were completed, each serum sample was designated either as Day 0 (pre-treatment) or Day 36 (post-treatment), with the final ILB^®^ treatment administered on Day 29.

An aliquot of each serum sample (500 µL) from either controls or patients with ALS was supplemented with 1.0 mL of HPLC-grade acetonitrile, vortexed for 60 s, centrifuged at 20,690× *g* for 15 min at 4 °C to precipitate proteins [10,11]. Supernatants were washed with large volumes of HPLC-grade chloroform to remove acetonitrile, centrifuged and the upper aqueous phases were transferred to different tubes, clearly labeled to identify the sample and stored at −80 °C until analyzed to determine different water-soluble compounds.

A second aliquot of 300 µL of each serum sample was protected from light and then processed to extract fat-soluble vitamins and antioxidants, using a method described in detail elsewhere [12]. Briefly, samples were supplemented with 1 mL of HPLC-grade acetonitrile, vortexed vigorously for 60 s and incubated at 37 °C for 1 h in a water bath under agitation, to maximize extraction of lipid soluble compounds. Samples were then centrifuged at 20,690× *g* for 15 min at 4 °C to remove precipitated proteins and the clarified supernatants stored at −80 °C until the HPLC analysis of fat-soluble vitamins and antioxidants.

In the aqueous phase of deproteinized serum samples, creatinine, uracil, β-pseudouridine, cytidine, hypoxhantine, xanthine, uric acid, uridine, inosine, guanosine, orotic acid, malondialdehyde (MDA), nitrite, nitrate, N-acetylaspartate (NAA) were separated and quantified by direct HPLC methods, with no sample derivatization [10,11,13]. Additionally, aspartate (ASP), glutamate (GLU), asparagine (ASN), serine (SER), glutamine (GLN), histidine (HIS), glycine (GLY), threonine (THR), citrulline (CITR), arginine (ARG), alanine (ALA), taurine (TAU), ɣ-aminobutyrate (GABA), tyrosine (TYR), S-adenosylhomocysteine (SAH), L-cystathionine (L-CYSTAT), valine (VAL), methionine (MET), tryptophan (TRP), phenylalanine (PHE), isoleucine (ILE), leucine (LEU), ornithine (ORN), lysine (LYS) were separated and quantified by HPLC using pre-column derivatization with orthophtalaldehyde (OPA) [14].

The following fat-soluble vitamins and antioxidants in deproteinized serum samples were separated and quantified by HPLC according to a method set up in our laboratory [12]: *all trans*-retinoic acid, *all trans*-retinol (vitamin A), α-tocopherol (vitamin E), ɣ-tocopherol, coenzyme Q_10_, astaxanthin, phytoene, lutein, zeaxanthin, *trans*-β-apo-8′-carotenal, β-cryptoxanthin, lycopene, α-carotene, β-carotene, violaxanthin, 25-hydroxycholecalciferol (vitamin D_3_).

All HPLC analyses were carried out using a Surveyor HPLC apparatus (Thermo Fisher Scientific, Rodano, Milan, Italy) equipped with a highly sensitive 5 cm light-path flow cell diode array UV detector, setup for acquisition between 200 and 550 nm wavelengths. Water-soluble compounds, free amino acids and amino group containing compounds were loaded (100 and 25 μL, respectively) onto a Hypersil C-18, 250 × 4.6 mm, 5 μm particle size column (Thermo Fisher Scientific, Rodano, Milan, Italy), while fat-soluble compounds (200 μL) were loaded onto a Hypersil Gold C-18, 200 × 4.6 mm, 5 μm particle size column (Thermo Fisher Scientific, Rodano, Milan, Italy). Both columns used were provided with their own guard columns. Data acquisition and analysis were performed using the ChromQuest^®^ software package provided by the HPLC manufacturer. Quantification of uracil, β-pseudouridine, cytidine, hypoxhantine, xanthine, uric acid, uridine, inosine, guanosine, orotic acid, malondialdehyde (MDA) was carried out at 260 nm wavelength [10,11,13]. Creatinine was quantified at 234 nm, whilst NAA, nitrite and nitrate were quantified at 206 nm wavelength [10,11,13]. OPA-amino acids and amino group-containing compound derivatives were quantified at 338 nm [14]. Lastly, fat-soluble vitamins and antioxidants were quantified at wavelengths between 260 and 500 nm [12].

To verify reproducibility of chromatographic runs, a mixture containing proper ultrapure standards with known concentrations was analyzed every other five serum samples.

Additionally, in all protein-free serum samples the concentration of lactate was determined spectrophotometrically using the method described by Artiss et al. [15].

### 2.7. Statistics

Comparison of the pre- and post-treatment subgroups was performed by the two-tailed Student’s *t*-test for paired samples. The comparison of each subgroup with the group of control healthy subjects was carried out by the one-way analysis of variance (ANOVA), followed by the Dunnett’s post-hoc test. Differences with *p* < 0.05 were considered statistically significant.

## 3. Results

### 3.1. Raw Data

Supplementary Tables S2–S4 report the raw data, means, standard deviations and *p*-values (significant results of the Student’s *t*-test are marked in red) of the circulating concentrations of metabolites found in patients with ALS. Only those compounds resulting in statistically significant differences on the pre-post ILB^®^ administration comparison (hypoxanthine, xanthine, uric acid, MDA, nitrite, nitrate, N-acetylaspartate, nitrite + nitrate and sum of oxypurines, Table S2; citrulline, alanine and ornithine/citrulline ratio, Table S3; α-tocopherol and γ-tocopherol, Table S4) are indicated. Similarly, Table S5 reports the serum levels of the aforementioned compounds recorded in the group of 163 age and sex matched healthy controls.

### 3.2. ILB^®^ Improves Patients’ Clinical Conditions and Decreases Neuronal Damage and Energy Metabolism Impairment

Data summarized in Table 2 show the effects of ILB^®^ treatment on the clinical parameters used for assessing symptom progression of ALS. At the end of the ILB^®^ administration period, a +8.6% increase in ALSFRS-R was observed (*p* < 0.001 compared to pre-treatment). Interestingly, at the time of blood withdrawal for the biochemical analyses of selected circulating metabolites (i.e., one week after the end of ILB^®^ treatment), clinical symptoms of patients with ALS displayed a further improvement leading to a final +13.3% overall increase in ALSFRS-R (*p* < 0.002 compared to pre-treatment).

**Figure 1:**
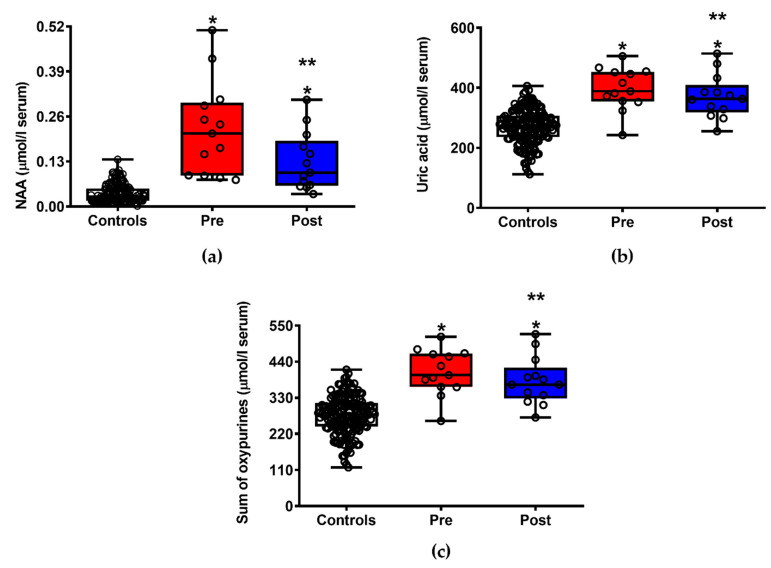
Box plots reporting minimum, maximum, median, 25% and 75% percentiles of the serum concentrations of the neuronal specific metabolite N-acetylaspartate (NAA, (**a**)) and of indices of energy metabolism impairment (uric acid, (**b**) and sum of oxypurines (**c**)) in 13 patients with ALS before (Pre) and after (Post) ILB^®^ treatment. The values found in a group of 163 healthy controls are also reported. (○): open circles are the values of metabolites in each subject enrolled in the study. Means ± S.D. of NAA in controls, patients with ALS before (Pre) and after (Post) ILB^®^ treatment were, respectively, 0.037 ± 0.026, 0.223 ± 0.136 and 0.130 ± 0.084 μmol/L serum. Means ± S.D. of uric acid in controls, patients with ALS before (Pre) and after (Post) ILB^®^ treatment were, respectively, 270.50 ± 57.90, 397.10 ± 70.32 and 371.40 ± 71.98 μmol/L serum. Means ± S.D. of sum of oxypurines in controls, patients with ALS before (Pre) and after (Post) ILB^®^ treatment were, respectively, 276.80 ± 58.45, 407.60 ± 69.34 and 381.20 ± 72.38 μmol/L serum. * Significantly different from Controls, *p* < 0.001. ** Significantly different from Pre, *p* < 0.05.

Figure 1 illustrates the changes in the circulating concentrations of NAA (a), uric acid (b) and sum of oxypurines (hypoxanthine + xanthine + uric acid) (c) recorded in patients with ALS before and after ILB^®^ administration. ILB^®^ treatment produced a significant reduction in ALS-related neuronal damage, as clearly evidenced by the 41.7% decrease (*p* < 0.05 compared to pre-treatment) of the serum levels of the neuron specific compound NAA. At the same time, ILB^®^-treated patients had decreased serum concentrations of uric acid (the final product of adenine nucleotide degradation) and the sum of oxypurines, suggesting an ILB^®^-induced amelioration of cell energy metabolism. It is worth underlining that, when compared with the group of healthy controls, patients with ALS, either before or after treatment, had significantly different values in any of the aforementioned compounds (*p* < 0.001).

### 3.3. ILB^®^ Decreases ALS-Related Oxidative/Nitrosative Stress

**Figure 2:**
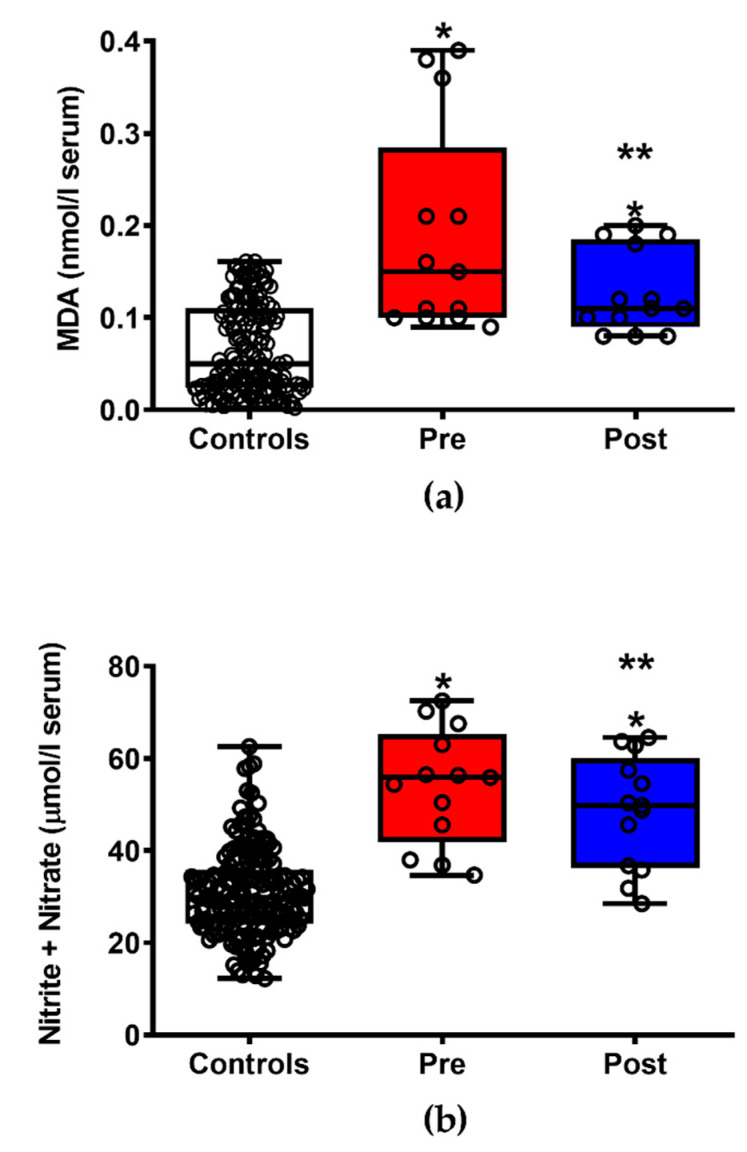
Box plots reporting minimum, maximum, median, 25% and 75% percentiles of the serum concentrations of lipid peroxidation end product (MDA, (**a**)) and stable compounds of nitric oxide metabolism (nitrite + nitrate, (**b**)) in 13 patients with ALS before (Pre) and after (Post) ILB^®^ treatment. The values measured in a group of 163 healthy controls are also reported. (○) Open circles are the values of metabolites in each subject enrolled in the study. Means ± S.D. of MDA in controls, patients with ALS before (Pre) and after (Post) ILB^®^ treatment were, respectively, 0.066 ± 0.048, 0.190 ± 0.114 and 0.128 ± 0.046 μmol/L serum. Means ± S.D. of nitrite + nitrate in controls, patients with ALS before (Pre) and after (Post) ILB^®^ treatment were, respectively, 30.90 ± 9.67, 53.98 ± 12.55 and 48.47 ± 12.25 μmol/L serum. * Significantly different from Controls, *p* < 0.001. ** Significantly different from Pre, *p* < 0.05.

As shown in Figure 2, patients with ALS after ILB^®^ treatment had significantly lower circulating biomarkers of both reactive oxygen species-mediated lipid peroxidation (a) (−33% in MDA values, *p* < 0.05) and of nitric oxide overproduction (b) (−10.2% in circulating nitrite + nitrate, *p* < 0.05), suggesting beneficial effects of the drug upon the mechanisms underlying insurgence of oxidative/nirosative stress. Notwithstanding the treatment effects, when compared with the group of healthy controls, patients with ALS, either before or after treatment, had significantly different values in both the aforementioned compounds (*p* < 0.001).

### 3.4. ILB^®^ Ameliorates ALS-Induced Changes of Serum Amino Acids

**Figure 3:**
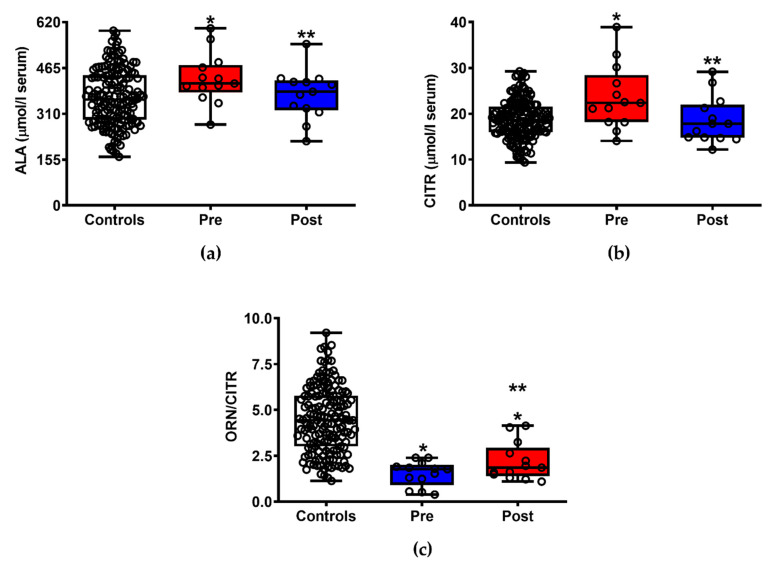
Box plots reporting minimum, maximum, median, 25% and 75% percentiles of the serum concentrations of amino acids related to muscular protein degradation (ALA, (**a**)) and to nitric oxide generation (CITR, (**b**) and ORN/CITR ratio, (**c**)) in patients with ALS before (Pre) and after (Post) ILB^®^ treatment. The values measured in a group of 163 healthy controls are also reported. (○) Open circles are the values of metabolites in each subject enrolled in the study. Means ± S.D. of ALA in controls, patients with ALS before (Pre) and after (Post) ILB^®^ treatment were, respectively, 364.40 ± 95.50, 429.00 ± 85.96 and 374.70 ± 83.43 μmol/L serum. Means ± S.D. of CITR in controls, patients with ALS before (Pre) and after (Post) ILB^®^ treatment were, respectively, 18.82 ± 4.22, 23.59 ± 7.03 and 18.60 ± 5.10 μmol/L serum. Means ± S.D. of ORN/CITR in controls, patients with ALS before (Pre) and after (Post) ILB^®^ treatment were, respectively, 4.47 ± 1.78, 1.52 ± 0.68 and 2−19 ± 1.03. * Significantly different from Controls, *p* < 0.001. ** Significantly different from Pre, *p* < 0.05.

Of the 25 amino acids and amino group-containing compounds that were quantified in serum samples of patients with ALS before and after ILB^®^ administrations, significant differences were found in the case of alanine (a) (−12.7%, *p* < 0.05), citrulline (b) (−21.2%, *p* < 0.05) and the ornithine/citrulline ratio (c) (+44%, *p* < 0.05), indicating an effect on muscular protein turnover and consistent with the significant decrease of nitric oxide production and counteraction of nitrosative stress(Figure 3). It is important to note that, in the case of alanine and citrulline, the administration of ILB^®^ was able to restore serum values equal to those found in healthy control subjects.

### 3.5. ILB^®^ Improves the Pattern of Circulating Fat-Soluble Antioxidants

**Figure 4:**
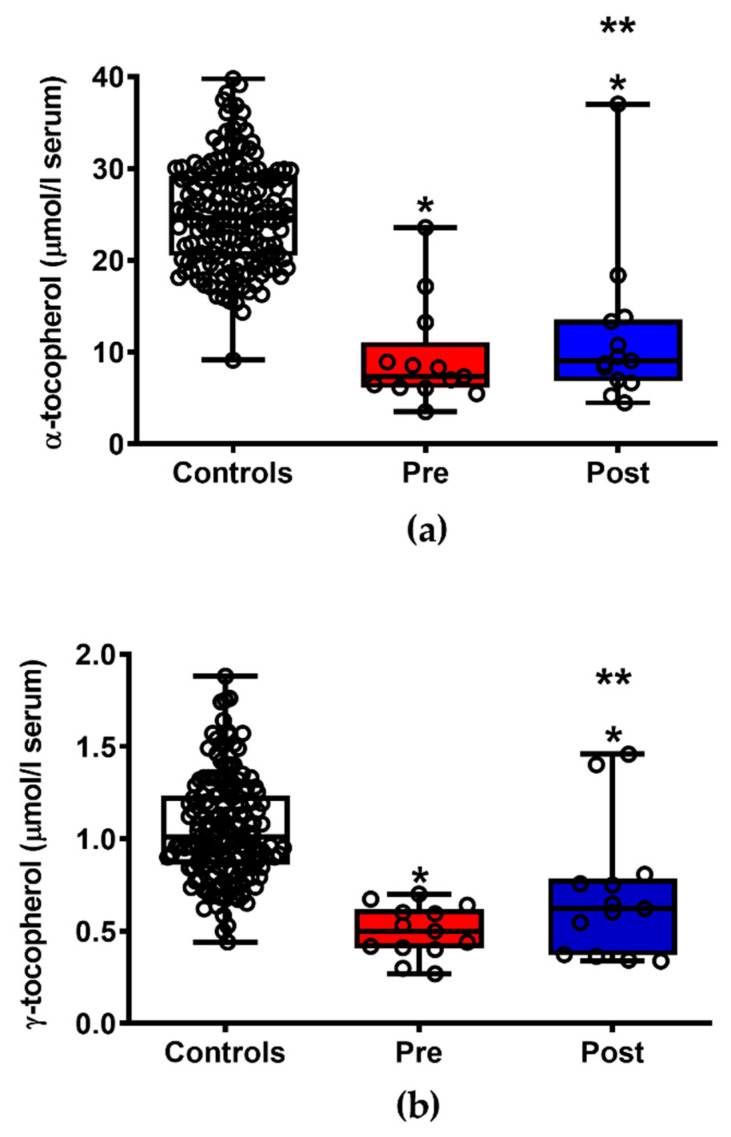
Box plots reporting minimum, maximum, median, 25% and 75% percentiles of the serum concentrations of the main vitamin E congeners (α-tocopherol, (**a**) and γ-tocopherol, (**b**)) in patients with ALS before (Pre) and after (Post) ILB^®^ treatment. The values measured in a group of 163 healthy controls are also reported. (○) Open circles are the values of metabolites in each subject enrolled in the study. Means ± S.D. of α-tocopherol in controls, patients with ALS before (Pre) and after (Post) ILB^®^ treatment were, respectively, 25.03 ± 5.78, 9.35 ± 5.54 and 11.70 ± 8.50 μmol/L serum. Means ± S.D. of γ-tocopherol in controls, patients with ALS before (Pre) and after (Post) ILB^®^ treatment were, respectively, 1.05 ± 0.28, 0.497 ± 0.139 and 0.692 ± 0.367 μmol/L serum. * Significantly different from Controls, *p* < 0.001. ** Significantly different from Pre, *p* < 0.05.

As shown in Figure 4, the group of 13 patients with ALS, one week after of drug administration, had significantly higher serum values of the most important fat-soluble antioxidant. In particular, serum levels of the two most abundant congeners of vitamin E found in the European diet, i.e., α-tocopherol (a) and γ-tocopherol (b), increased by 21.5% and 39.2% (*p* < 0.05), respectively, thus conferring a better protection to the unsaturated fatty acids of membrane phospho-lipids that are the main target of reactive oxygen species-mediated lipid peroxidation. However, both compounds were significantly lower than the values measured in healthy controls, either before or after drug administration (*p* < 0.001).

## 4. Discussion

The relevance of metabolic dysfunction to acute and chronic neurodegeneration has clearly been established in the last decade [16,17]. ALS has also been included in the list of chronic, progressive neurodegenerative disorders in which metabolic alterations, particularly those occurring to the so-called “central metabolism” [18,19], have been indicated not only as biochemical signatures of the disease [20] but also as molecular mechanisms connected to its pathogenesis and progression [21,22]. Hence, drug treatments capable of improving ALS-induced metabolic dysfunctions are considered as potentially promising therapies to be investigated in this still “orphan drug disease” [23,24]. Results reported in the present clinical trial strongly suggest that ILB^®^ administration produces metabolic benefits that are translated into evident improvements in clinical outcome for patients with ALS.

When evaluating the results of the targeted metabolic analyses, in serum from 13 patients with ALS (before and one week after 29 days of ILB^®^ administration), of the 55 quantified compounds that are connected to energy metabolism, mitochondrial function, oxidative/nitrosative stress, water- and fat-soluble antioxidant defenses, one of the most remarkable differences occurred with the levels of NAA. In accordance with the data of Simone et al. [25], we found that patients with ALS had nearly five-times higher values of circulating NAA than those of control healthy subjects. Although NAA serum levels after ILB^®^ treatment were still 2.4 times higher than the values recorded in controls, a 41.7% decrease compared to pre-treatment levels was detected. Previous clinical studies have demonstrated that brain levels of NAA in patients with ALS are significantly lower than those of healthy subjects [26,27,28] and correlates negatively with time of patients’ survival [29]. Furthermore, in a mouse model of selective motoneuronal loss, mimicking the primary pathology associated with ALS, we have very recently found a dramatic depletion of NAA in spinal cord extracts [30]. It has been shown that changes in cerebral NAA levels under pathological conditions are not simply due to neuronal loss but may also occur under conditions of mitochondrial dysfunction with consequent energy penalty [31,32,33,34,35]. Therefore, it is tempting to speculate that lower serum NAA concentrations in the cohort of patients with ALS following ILB^®^ administration was due to improved cerebral mitochondrial metabolism. This is supported by recent evidence showing that ILB^®^ administration dose-dependently restores brain energy metabolism and NAA concentrations following severe traumatic brain injury in an animal model in vivo [6].

In accordance with this hypothesis, we found that patients with ALS after ILB^®^ treatment had decreased serum levels of both uric acid and sum of oxypurines (hypoxanthine + xanthine + uric acid). These compounds, particularly uric acid, represent the end products of the adenine nucleotide degradation pathway occurring at a high rate under conditions of impaired energy metabolism, i.e., when imbalance between ATP production and consumption takes place [36,37]. Increased oxypurines have been observed during coronary bypass surgery [38], following traumatic brain injury [39], in patients affected by multiple sclerosis (correlating with disease progression, clinical subtype and neuroradiological findings) [40,41], as well as under various experimental conditions of cellular energy penalty [42,43,44] including those found in muscle tissue in an ALS-like murine model of motoneuron ablation [30]. Recently, using ^31^P-magnetic resonance spectroscopy for the quantification of energy-related metabolites in vivo, it has been found that patients with ALS have evident mitochondrial dysfunction in both brain and muscle tissues [45]. Therefore, the increase in serum uric acid and sum of oxypurines evident in the patients with ALS, compared to levels in healthy controls, may represent a biochemical signature of impaired neuronal and muscle metabolism that can be attenuated successfully by the treatment with ILB^®^.

Numerous pathological conditions characterized by protracted mitochondrial dysfunction are accompanied by increased production of reactive oxygen (ROS) and reactive nitrogen species (RNS) triggering the insurgence of oxidative/nitrosative stress [46,47,48,49]. If for ROS overproduction mitochondrial malfunctioning is certainly imputed as the main cause [50], in the case of excessive RNS formation the main origin is linked to overexpression of inducible nitric oxide synthase (iNOS) with a consequent high rate of nitric oxide generation [51], often mediated by (neuro)inflammatory processes [52,53]. In preclinical and clinical studies of ALS, clear evidence of ROS and RNS-mediated damages have previously been shown [54,55,56,57] accompanied by a decrease in brain and muscle concentrations of reduced glutathione [58,59], i.e., one of the most important intracellular water-soluble antioxidant and scavenger of excessive formation of nitric oxide and RNS [60]. In the cohort of patients with ALS, we found that both MDA (quantified by direct HPLC method with no derivatization) and the sum of nitrite + nitrate were, before treatment, significantly higher than the values recorded in healthy controls. Although ILB^®^ administration did not abolish evidence of circulating oxidative/nitrosative stress associated with ALS, a significant decrease in both parameters was evident one week after the 29 days period of drug treatment. Additionally, patients with ALS post ILB^®^ treatment had significantly higher serum levels of the main fat-soluble antioxidants (the α- and γ-congeners of tocopherol) that may confer better protection to unsaturated fatty acids of biological membrane phospholipids towards ROS-mediated lipid peroxidation It is worth underlining that ILB^®^ displayed, under different experimental conditions, remarkable anti-inflammatory activity [5] and capacity to diminish oxidative/nitrosative stress [6].

The last beneficial effects of the ILB^®^ administration to this cohort of patients with ALS were changes in serum levels of ALA, CITR and the ORN/CITR ratio when compared with the corresponding pre-treatment levels. In the pre-treatment samples, these parameters were significantly different from those of controls (higher ALA and CITR, and lower ORN/CITR ratio), suggesting higher degradation rate of muscular proteins (ALA) and confirming higher nitric oxide production through higher activity of the iNOS enzymes (CITR and ORN/CITR ratio). Previous data indicate various anomalies in the tissue and serum levels of free amino acids [61,62,63,64], supporting the notion of a profound metabolic derangement induced by ALS. The benefits of ILB^®^ treatment on these parameters confirm the data obtained with sum of nitrite + nitrate and suggest that the drug may affect positively muscle metabolism perhaps either through its anti-inflammatory activities [5] or through its capacity to induce an amelioration of energy-related metabolism and of amino acid metabolism dysregulation under conditions of cell sufferance [6].

In conclusion, although obtained in a restricted number of patients (not allowing to evaluate potential age and/or sex differences of the serum biochemical analyses) this longitudinal study produced very encouraging results concerning the effects of ILB^®^ administration to patients suffering from ALS. As there are no current effective drug treatments licensed for clinical use other than riluzole that has a very limited benefit, the present findings indicate the utility of pharmacological interventions positively acting on the ALS-induced metabolic changes, specifically on energy-related mitochondrial functions, oxidative/nitrosative stress and amino acid metabolism. Results from further studies with larger patient numbers are needed.

## Figures and Tables

**Table 1:** Coding of the serum samples.

Sample Number	Subject Number and Sampling Time
1	Subject 101, Day 0
2	Subject 101, Day 36
3	Subject 102, Day 0
4	Subject 10, Day 36
5	Subject 103, Day 0
6	Subject 103, Day 36
7	Subject 104, Day 0
8	Subject 104, Day 36
9	Subject 105, Day 0
10	Subject 105, Day 36
11	Subject 106, Day 0
12	Subject 106, Day 36
13	Subject 107, Day 0
14	Subject 107, Day 36
15	Subject 108, Day 0
16	Subject 108, Day 36
17	Subject 109, Day 0
18	Subject 109, Day 36
19	Subject 110, Day 0
20	Subject 110, Day 36
21	Subject 111, Day 0
22	Subject 111, Day 36
23	Subject 112, Day 0
24	Subject 112, Day 36
25	Subject 113, Day 0
26	Subject 113, Day 36

**Table 2:** Change in ALS patient functional rating over the ILB^®^ treatment period assessed by ALSFRS-R.

Day	ALSFRS-R ± SD
0 (Pre-treatment)	36.1 ± 6.7
29 (Final treatment)	39.2 ± 6.3 *
36 (Post-treatment)	40.9 ± 6.9 **

* Significantly different compared to day 0, *p* < 0.001. ** Significantly different compared to day 0, *p* < 0.002.

## Data Availability

The underpinning data that support the findings in this study are available from the EU Clinical Trials Register (https://www.clinicaltrialsregister.eu/ctr-search/trial/2017-005065-47/results, accessed on 5 September 2020).

## Supplementary Materials

The following are available online at https://www.mdpi.com/article/10.3390/jpm11080794/s1, Table S1: Clinical trial protocol, Table S2: Raw data reporting the circulating concentrations of hypoxanthine (HYP), xanthine (XAN), uric acid, malondialdehyde (MDA), nitrite (-NO_2_), nitrate (-NO_3_), N-acetylaspartate (NAA), nitrite + nitrate and sum of oxypurines (hypoxanthine + xanthine + uric acid), detected in serum of each ALS patient included in the study, before and after ILB^®^ administration, Table S3: Raw data reporting the circulating concentrations of citrulline (CITR), alanine (ALA), ornithine/citrulline ratio (ORN/CITR) detected in serum of each ALS patient included in the study, before and after ILB^®^ administration, Table S4: Raw data reporting the circulating concentrations of the two main congeners of vitamin E (α-tocopherol and γ-tocopherol) detected in serum of each ALS patient included in the study, before and after ILB^®^ administration, Table S5: Raw data reporting the circulating concentrations of the relevant metabolites detected in serum of heathy controls.
