# Supplementary material for: ILB® Attenuates Clinical Symptoms and Serum Biomarkers of Oxidative/Nitrosative Stress and Mitochondrial Dysfunction in Patients with Amyotrophic Lateral Sclerosis
Source: J Pers Med. 2021 Aug 14;11(8):794. doi: 10.3390/jpm11080794 (PMC8399678; doi:10.3390/jpm11080794)
Supplement: Supplementary file 1 [file jpm-11-00794-s001.zip › jpm-1291257-supplementary.pdf]

Table S1. Clinical trial protocol

**Study Title**<br><br>A single-centre, open single-arm study where the safety, tolerability and efficacy of subcutaneously administered ILB® will be evaluated in patients with Amyotrophic Lateral Sclerosis	
**Study code**<br><br>TM-105	**EudraCT No**<br><br>2017-005065-47
**Study period**<br><br>Estimated date of first subject enrolled: Q2 2018<br><br>Estimated date of last subject completed: Q3 2019	**Phase of development**<br><br>Phase IIa - pilot
**Study design and duration**<br><br>This was a Phase IIa single-centre, open single-arm study in patients with Amyotrophic Lateral Sclerosis.<br><br>Eligible subjects were administered weekly doses of 1 mg/kg ILB®. A total of 5 subcutaneous (s.c.) doses at the study clinic. The study consisted of 10 visits; One 2-part screening visit, 5 IMP administration visits, and 3 follow-up visits. Each individual patient's study participation was approximately 4 months, including the screening and follow-up visits.	
**Number of patients planned:**<br><br>15 patients	
**Objectives and endpoints**<br><br>*Primary objective:*<br><br>The primary objective of the study was to evaluate the safety and tolerability of ILB® in patients diagnosed with ALS.<br><br>*Primary endpoints:*<br><br>Frequency, seriousness and intensity of Treatment-emergent Adverse Events (TEAEs)<br><br>Physical examination<br><br>Vital signs<br><br>Electrocardiogram (ECG) recordings	

Safety laboratory measurements: clinical chemistry, haematology including activated partial thromboplastin time (APTT)

Secondary objective:

The secondary objective of the study was to evaluate efficacy of ILB® in patients diagnosed with ALS.

Secondary endpoints:

Functional rating with ALS Functional Rating Scale – Revised (ALSFRS-R)

Functional rating with Norris scale

Evaluation of ALS biomarker Neurofilament Light chain (NFL)

Evaluation of biomarkers for neurological disorders (see 11.4.2)

Pulmonary function (FVC)

Quality of Life (QoL)

Autonomous symptoms

Levels of Hepatocyte Growth Factor (HGF)

Pharmacokinetic measurements of ILB® in plasma

Changes in APTT (effect APTT)

Exploratory objectives:

To evaluate the effect of ILB® on levels of *e.g.* growth factors, cytokines and biomarkers in plasma and cerebrospinal fluid (CSF).

Safety and efficacy assessments

Safety:

AE assessments started at administration of the first dose of ILB®.

A physical exam was obtained at all visits. On the days of ILB® administration (Visit 2 to Visit 6), physical examination was obtained before dosing.

Vital signs (blood pressure, heart rate and body temperature) were obtained at all visits.

12-lead ECG were obtained at Visit 1 and Visit 7.

Lab tests (clinical chemistry, haematology, haemostatis) were obtained at all visits. On the days of ILB® administration (Visit 2 to Visit 6), lab tests were obtained before dosing.

Efficacy:

The clinical investigator completed the ALS Functional Rating Scale - Revised (ALSFRS-R) at all visits. On the days of ILB® administration, rating scales were completed before dosing.

The clinical investigator completed the Norris functional rating scales at all visits. On the days of ILB® administration, rating scales were completed before dosing.

Blood and cerebrospinal fluid (CSF) samples for evaluation of the ALS biomarker Neurofilament Light chain (NFL) were obtained at Visit 1 after eligibility was confirmed and at Visit 7.

Blood and CSF samples for analysis of biomarkers for neurological disorders were obtained at Visit 1 after eligibility is confirmed and at Visit 7.

Respiratory function (FVC) was evaluated at all visits. On the days of ILB® administration (Visit 2 to Visit 6), respiratory function was assessed before dosing.

Quality of Life (QoL) was evaluated using Visual Analogue Scale (VAS)-based questionnaire filled out by patient and, if applicable, a next of kin, at Visit 1, Visit 2, Visit 4, Visit 6 and Visit 8.

Autonomous symptoms were evaluated according to a 4-grade scale for a range of parameters at all visits.

Levels of Hepatocyte Growth Factor (HGF), pharmacokinetic measurements and effect APTT were obtained in conjunction with the first and last ILB® administration (Visit 2 and Visit 6). Blood samples were obtained pre-dose, 30 minutes and 1, 2, 2.5, 3, 4, 6 hours after dosing.

Eligibility criteria

Inclusion criteria:

Willing and able to give written informed consent for participation in the study.

Clinical diagnosis of Amyotrophic Lateral Sclerosis.

Male or female patients between 18 to 80 years of age (inclusive).

Forced Vital Capacity (FVC) ≥ 65% of predicted value for gender, height and age at screening.

Evaluated with ALSFRS-R and Norris clinical rating scales for at least the past 4 weeks before study drug administration.

Exclusion criteria:

Unable to understand information about the study or are expected not to collaborate with the study team.

Concurrent serious disease, other than ALS, at the discretion of the Investigator.

Pregnancy.

Patients of child-bearing potential not willing to use adequate double contraception with < 1% failure rate after the screening visit until the last visit.

Addiction to drugs or alcohol.

Confirmed HIV, Hepatitis B or C.

Known bleeding disorders or abnormal bleeding events.

Treatment with anticoagulant drugs warfarin and novel oral anticoagulants (NOAC) within the last 14 days prior to screening.

Treatment with Riluzole or Lamotrigine within the last 28 days prior to study drug administration.

Hypersensitivity to dextran sulfate.

Poor venous access.

Patients with clinically significant abnormal PK-INR, fibrinogen, von Willebrand factor and activated partial thromboplastin time (APTT) at screening.

Investigational Medicinal Product (IMP), dosage and mode of administration

The active pharmaceutical ingredient of the IMP was a LMW-DS, named ILB®, that has a mean molecular weight of 5 kDa with 20% sulphation. ILB® is a unique and distinct LMW-DS formulation whose structure, formulation, synthesis and structure has been previously described in detail in a published patent document (publication number: WO 2016/076780 – New dextran sulphate). ILB® was provided by Tikomed AB in 10 mL vials containing a solution of 20 mg/mL ILB® in 9 mg/mL NaCl. A single batch of drug was used throughout the study (batch #8059701).

ILB® was injected subcutaneously on alternating sides of the abdomen. Five injections of 1 mg/kg, with a weekly dosing interval, were administered. The exact dose administered depended on the patient's body weight at Visit 2 (Day 1), prior to the first ILB® administration.

Statistical methods

No formal sample size calculation was performed for this study. The proposed sample size was considered sufficient in this early phase II development to provide adequate information on the patients.

Categorical data was presented as counts and percentages. Individual patient data was listed by subject number and, where applicable, assessment time.

Continuous data were summarised using descriptive statistics. The number of observations, mean, median, maximum, minimum and standard deviation were reported. Individual patient data was listed by subject number and, where applicable, assessment time.

Individual plasma concentration-time data of ILB® were evaluated using a validated Phoenix WinNonlin® software. The PK parameters were calculated by non-compartmental analysis (NCA). Descriptive statistics for the assessed PK parameters were presented for each parameter and first and last ILB® administration (Visit 2 and Visit 6), as well as pooled data over the dosing days (if appropriate).

A more technical and detailed elaboration of the principal features was presented in a separate Statistical Analysis Plan (SAP).

Table S2. Raw data reporting the circulating concentrations of hypoxanthine (HYP), xanthine (XAN), uric acid, malondialdehyde (MDA), nitrite (NO2), nitrate (NO3), N-acetylaspartate (NAA), nitrite + nitrate and sum of oxypurines (hypoxanthine + xanthine + uric acid), detected in serum of each patient included in the study, before and after ILB® administration. Values are expressed as µmol/l serum. Significant differences on the Student's t-test for paired samples are marked in red. The concentrations of creatinine, pyrimidines (uracil, β-pseudouridine, cytidine, uridine), purine nucleosides (inosine and guanosine), lactate and IL-6 did not differ before and after treatment and were omitted from the Table for the sake of clarity.

PATIENT ID	HYP	XAN	URIC ACID	MDA	NO2	NO3	NAA	SUM OXYPURINES	NO3 + NO2
**TM - 105 SUBJ 101 EXPL SERUM B VISIT 1B**	9.023	1.229	505.600	0.160	2.715	60.265	0.169	515.852	62.980
**TM - 105 SUBJ 102 EXPL SERUM D VISIT 1B**	9.245	1.625	454.547	0.360	2.546	67.713	0.427	465.417	70.259
**TM - 105 SUBJ 103 EXPL SERUM A VISIT 1B**	7.069	2.005	446.250	0.110	8.111	59.439	0.309	455.324	67.550
**TM - 105 SUBJ 104 EXPL SERUM A VISIT 1B**	10.565	1.379	324.284	0.390	3.816	34.168	0.509	336.228	37.984
**TM - 105 SUBJ 105 EXPL SERUM B VISIT 1B**	14.524	1.453	243.031	0.210	2.181	53.689	0.083	259.008	55.870
**TM - 105 SUBJ 106 EXPL SERUM A VISIT 1B**	8.632	1.303	388.884	0.210	3.490	52.949	0.251	398.819	56.439
**TM - 105 SUBJ 107 EXPL SERUM A VISIT 1B**	7.943	0.895	382.110	0.100	4.836	45.586	0.291	390.948	50.422
**TM - 105 SUBJ 108 EXPL SERUM B VISIT 1B**	9.694	0.944	451.750	0.380	6.158	66.314	0.211	462.388	72.472
**TM - 105 SUBJ 109 EXPL SERUM A VISIT 1B**	8.280	1.533	416.884	0.150	2.873	34.001	0.151	426.697	36.874
**TM - 105 SUBJ 110 EXPL SERUM A VISIT 1B**	5.852	0.732	356.610	0.110	3.233	51.129	0.089	363.194	54.362
**TM - 105 SUBJ 111 EXPL SERUM A VISIT 1B**	8.304	0.946	352.960	0.100	2.545	32.144	0.090	362.210	34.689
**TM - 105 SUBJ 112 EXPL SERUM A VISIT 1B**	11.405	1.175	372.368	0.090	4.814	40.750	0.077	384.948	45.564
**TM - 105 SUBJ 113 EXPL SERUM B VISIT 1B**	9.280	1.075	467.360	0.100	3.567	52.760	0.237	477.715	56.327
**Mean**	**9.217**	**1.253**	**397.126**	**0.190**	**3.914**	**50.070**	**0.223**	**407.596**	**53.984**
**S.D.**	**2.134**	**0.350**	**70.317**	**0.114**	**1.692**	**12.028**	**0.136**	**69.339**	**12.555**
**Two-tailed t-test for paired samples**	**0.266**	**0.298**	**0.046**	**0.038**	**0.905**	**0.044**	**0.013**	**0.042**	**0.042**

PATIENT ID	HYP	XAN	URIC ACID	MDA	NO2	NO3	NAA	SUM OXYPURINES	NO3 + NO2
**TM - 105 SUBJ 101 EXPL SERUM D VISIT 7**	11.926	1.812	480.219	0.190	6.043	39.504	0.207	493.957	45.547
**TM - 105 SUBJ 102 EXPL SERUM D VISIT 7**	8.700	1.436	386.179	0.180	2.226	61.420	0.250	396.315	63.646
**TM - 105 SUBJ 103 EXPL SERUM D VISIT 7**	7.770	2.003	514.207	0.100	7.389	55.348	0.098	523.980	62.737
**TM - 105 SUBJ 104 EXPL SERUM A VISIT 7**	7.799	0.887	298.991	0.190	3.800	28.008	0.152	307.677	1.100
**TM - 105 SUBJ 105 EXPL SERUM B VISIT 7**	12.835	1.073	255.726	0.080	1.699	62.858	0.055	269.634	64.557
**TM - 105 SUBJ 106 EXPL SERUM D VISIT 7**	6.024	0.741	385.095	0.200	2.332	46.404	0.073	391.860	48.736
**TM - 105 SUBJ 107 EXPL SERUM B VISIT 7**	7.072	0.890	338.305	0.110	3.899	50.621	0.308	346.267	54.520
**TM - 105 SUBJ 108 EXPL SERUM D VISIT 7**	11.513	1.170	432.982	0.120	3.985	53.432	0.088	445.665	57.417
**TM - 105 SUBJ 109 EXPL SERUM A VISIT 7**	7.677	1.240	360.886	0.110	2.121	26.359	0.062	369.803	28.480
**TM - 105 SUBJ 110 EXPL SERUM D VISIT 7**	5.770	0.751	363.353	0.080	5.649	44.702	0.058	369.874	50.351
**TM - 105 SUBJ 111 EXPL SERUM D VISIT 7**	7.475	0.769	329.169	0.080	3.169	33.522	0.036	337.413	36.691
**TM - 105 SUBJ 112 EXPL SERUM D VISIT 7**	9.172	0.935	307.782	0.120	6.109	43.643	0.125	317.889	49.752
**TM - 105 SUBJ 113 EXPL SERUM D VISIT 7**	9.112	1.324	374.970	0.100	1.759	34.045	0.173	385.406	35.804
**Mean**	**8.680**	**1.156**	**371.374**	**0.128**	**3.860**	**44.605**	**0.130**	**381.211**	**48.465**
**S.D.**	**2.207**	**0.403**	**71.979**	**0.045**	**1.895**	**11.966**	**0.084**	**72.384**	**12.246**

Table S3. Raw data reporting the circulating concentrations of citrulline (CITR), alanine (ALA), ornithine/citrulline ratio (ORN/CITR) detected in serum of each patient included in the study, before and after ILB® administration. Values are expressed as μmol/l serum. Significant differences on the Student's t-test for paired samples are marked in red. The concentrations of other amino acids and amino group-containing compounds (aspartate, glutamate, asparagine, serine, glutamine, histidine, glycine, threonine, arginine, taurine, γ-aminobutyrate, tyrosine, S-adenosylhomocysteine, L-cystathionine, valine, methionine, tryptophan, phenylalanine, isoleucine, leucine, ornithine, lysine) did not differ before and after treatment and were omitted from the Table for the sake of clarity.

PATIENT ID	CITR	ALA	ORN/CITR	PATIENT ID	CITR	ALA	ORN/CITR
**TM - 105 SUBJ 101 EXPL SERUM B VISIT 1B**	14.082	412.563	2.399	**TM - 105 SUBJ 101 EXPL SERUM D VISIT 7**	17.824	217.393	1.312
**TM - 105 SUBJ 102 EXPL SERUM D VISIT 1B**	32.910	428.911	1.312	**TM - 105 SUBJ 102 EXPL SERUM D VISIT 7**	14.863	416.915	1.859
**TM - 105 SUBJ 103 EXPL SERUM A VISIT 1B**	21.110	432.289	2.101	**TM - 105 SUBJ 103 EXPL SERUM D VISIT 7**	14.701	428.179	2.645
**TM - 105 SUBJ 104 EXPL SERUM A VISIT 1B**	21.239	484.059	1.854	**TM - 105 SUBJ 104 EXPL SERUM A VISIT 7**	17.852	429.757	1.228
**TM - 105 SUBJ 105 EXPL SERUM B VISIT 1B**	22.373	562.368	0.560	**TM - 105 SUBJ 105 EXPL SERUM B VISIT 7**	18.955	546.009	1.100
**TM - 105 SUBJ 106 EXPL SERUM A VISIT 1B**	18.152	403.358	0.523	**TM - 105 SUBJ 106 EXPL SERUM D VISIT 7**	13.831	328.553	2.235
**TM - 105 SUBJ 107 EXPL SERUM A VISIT 1B**	24.141	273.188	0.391	**TM - 105 SUBJ 107 EXPL SERUM B VISIT 7**	26.803	267.262	1.596
**TM - 105 SUBJ 108 EXPL SERUM B VISIT 1B**	38.921	466.325	1.249	**TM - 105 SUBJ 108 EXPL SERUM D VISIT 7**	29.179	315.148	1.490
**TM - 105 SUBJ 109 EXPL SERUM A VISIT 1B**	18.214	405.240	1.798	**TM - 105 SUBJ 109 EXPL SERUM A VISIT 7**	12.194	408.546	4.132
**TM - 105 SUBJ 110 EXPL SERUM A VISIT 1B**	22.540	346.071	1.908	**TM - 105 SUBJ 110 EXPL SERUM D VISIT 7**	22.693	374.396	1.626
**TM - 105 SUBJ 111 EXPL SERUM A VISIT 1B**	30.164	399.005	1.535	**TM - 105 SUBJ 111 EXPL SERUM D VISIT 7**	21.249	384.615	1.959
**TM - 105 SUBJ 112 EXPL SERUM A VISIT 1B**	26.660	598.715	1.777	**TM - 105 SUBJ 112 EXPL SERUM D VISIT 7**	14.447	417.786	4.043
**TM - 105 SUBJ 113 EXPL SERUM B VISIT 1B**	16.194	365.052	2.396	**TM - 105 SUBJ 113 EXPL SERUM D VISIT 7**	19.894	336.769	3.253
**Mean**	**23.592**	**429.011**	**1.523**	**Mean**	**18.807**	**374.718**	**2.191**
**S.D.**	**7.028**	**85.956**	**0.683**	**S.D.**	**5.128**	**83.428**	**1.031**
**Two-tailed t-test for paired samples**	**0.021**	**0.022**	**0.037**				

Table S4. Raw data reporting the circulating concentrations of the two main congeners of Vitamin E (α-tocopherol and γ-tocopherol) detected in serum of each patient included in the study, before and after ILB® administration. Values are expressed as μmol/l serum. Significant differences on the Student's t-test for paired samples are marked in red. The concentrations of other fat-soluble vitamins and antioxidants (*all trans*-retinoic acid, *all trans*-retinol, coenzyme Q_{10}, astaxanthin, phytoene, lutein, zeaxanthin, *trans*-β-apo-8′-carotenal, β-cryptoxanthin, lycopene, α-carotene, β-carotene, violaxanthin, 25-hydroxycholecalciferol) did not differ before and after treatment and were omitted from the Table for the sake of clarity.

PATIENT ID	α-tocopherol	γ-tocopherol	PATIENT ID	α-tocopherol	γ-tocopherol
**TM - 105 SUBJ 101 EXPL SERUM B VISIT 1B**	8.909	0.498	**TM - 105 SUBJ 101 EXPL SERUM D VISIT 7**	17.824	217.393
**TM - 105 SUBJ 102 EXPL SERUM D VISIT 1B**	6.419	0.640	**TM - 105 SUBJ 102 EXPL SERUM D VISIT 7**	14.863	416.915
**TM - 105 SUBJ 103 EXPL SERUM A VISIT 1B**	6.978	0.529	**TM - 105 SUBJ 103 EXPL SERUM D VISIT 7**	14.701	428.179
**TM - 105 SUBJ 104 EXPL SERUM A VISIT 1B**	7.314	0.297	**TM - 105 SUBJ 104 EXPL SERUM A VISIT 7**	17.852	429.757
**TM - 105 SUBJ 105 EXPL SERUM B VISIT 1B**	8.283	0.593	**TM - 105 SUBJ 105 EXPL SERUM B VISIT 7**	18.955	546.009
**TM - 105 SUBJ 106 EXPL SERUM A VISIT 1B**	6.137	0.410	**TM - 105 SUBJ 106 EXPL SERUM D VISIT 7**	13.831	328.553
**TM - 105 SUBJ 107 EXPL SERUM A VISIT 1B**	6.053	0.672	**TM - 105 SUBJ 107 EXPL SERUM B VISIT 7**	26.803	267.262
**TM - 105 SUBJ 108 EXPL SERUM B VISIT 1B**	3.504	0.400	**TM - 105 SUBJ 108 EXPL SERUM D VISIT 7**	29.179	315.148
**TM - 105 SUBJ 109 EXPL SERUM A VISIT 1B**	5.459	0.600	**TM - 105 SUBJ 109 EXPL SERUM A VISIT 7**	12.194	408.546
**TM - 105 SUBJ 110 EXPL SERUM A VISIT 1B**	8.528	0.417	**TM - 105 SUBJ 110 EXPL SERUM D VISIT 7**	22.693	374.396
**TM - 105 SUBJ 111 EXPL SERUM A VISIT 1B**	13.223	0.438	**TM - 105 SUBJ 111 EXPL SERUM D VISIT 7**	21.249	384.615
**TM - 105 SUBJ 112 EXPL SERUM A VISIT 1B**	23.568	0.268	**TM - 105 SUBJ 112 EXPL SERUM D VISIT 7**	14.447	417.786
**TM - 105 SUBJ 113 EXPL SERUM B VISIT 1B**	17.150	0.700	**TM - 105 SUBJ 113 EXPL SERUM D VISIT 7**	19.894	336.769
**Mean**	9.348	0.497	**Mean**	**18.807**	**374.718**
**S.D.**	5.544	0.139	**S.D.**	**5.128**	**83.428**
**Two-tailed t-test for paired samples**	0.037	0.028			

Table S5. Raw data reporting the circulating concentrations in serum of heathy controls of those metabolites affected by ILB treatment in patients with ALS. Values are expressed as μmol/l serum. Means and S.D. are indicated at the end of each column and marked in red.

HEALTHY CONTROLS	URIC ACID	MDA	NAA	SUM OXYPURINES	NO2 + NO3	CITR	ALA	ORN	ORN/CITR	α-tocopherol	γ-tocopherol
1	281.680	0.026	0.077	286.180	26.960	25.215	468.360	58.240	2.310	22.480	0.498
2	193.380	0.018	0.013	198.630	42.940	14.640	340.080	64.747	4.423	25.660	1.291
3	178.800	0.036	0.054	188.750	29.740	14.040	199.800	32.427	2.310	17.810	1.009
4	227.580	0.090	0.026	233.420	35.980	20.670	321.480	102.400	4.954	31.020	1.489
5	280.530	0.024	0.043	285.950	33.020	15.825	357.840	100.213	6.333	19.590	1.288
6	350.900	0.028	0.031	362.660	33.770	20.370	398.880	91.200	4.477	24.550	1.233
7	276.900	0.014	0.037	282.210	37.980	19.875	366.720	69.387	3.491	15.900	1.080
8	346.890	0.054	0.024	357.360	45.520	21.960	417.120	86.827	3.954	33.310	0.980
9	246.410	0.029	0.067	252.580	35.720	21.765	292.440	98.240	4.514	19.150	1.350
10	359.330	0.009	0.032	370.710	35.250	22.095	272.760	64.053	2.899	18.490	1.280
11	250.720	0.078	0.054	258.480	33.710	25.000	431.040	157.893	6.316	24.450	0.749
12	345.110	0.080	0.028	348.620	30.090	19.875	289.220	67.520	3.397	32.240	0.960
13	359.160	0.011	0.094	370.360	46.830	18.000	227.760	138.187	7.677	27.090	1.560
14	301.060	0.009	0.049	309.400	31.650	23.445	311.520	81.120	3.460	25.370	1.330
15	380.900	0.020	0.016	389.170	39.600	19.980	340.240	36.880	1.846	27.760	1.490
16	258.700	0.038	0.016	263.530	34.680	15.000	314.400	125.153	8.344	39.110	1.640
17	292.360	0.033	0.058	300.510	32.860	19.875	440.400	131.893	6.636	20.420	1.310
18	300.520	0.005	0.015	310.010	32.530	18.000	369.120	81.320	4.518	22.550	1.880
19	317.040	0.004	0.035	326.410	38.660	23.445	344.760	84.327	3.597	20.760	1.460
20	320.470	0.035	0.015	326.890	18.900	19.980	470.760	28.640	1.433	16.560	0.770
21	181.600	0.019	0.025	185.520	17.860	22.650	368.880	100.453	4.435	9.120	0.920
22	307.040	0.038	0.051	313.930	39.800	23.235	273.840	94.907	4.085	20.520	0.800
23	291.560	0.037	0.040	295.640	20.870	22.350	301.170	97.400	4.358	30.120	1.030
24	239.170	0.050	0.025	243.240	34.670	15.435	495.960	43.687	2.830	24.240	1.100
25	276.890	0.006	0.021	287.400	22.950	16.000	438.960	52.160	3.260	24.600	0.720
26	331.970	0.047	0.045	339.990	47.490	23.040	356.440	44.693	1.940	23.520	0.690
27	267.090	0.033	0.016	275.110	33.260	16.000	459.120	56.447	3.528	17.880	0.845
28	297.160	0.011	0.041	306.400	20.610	15.330	366.600	116.800	7.619	21.720	0.960
29	336.400	0.032	0.026	345.490	32.890	28.875	469.320	43.327	1.500	26.160	1.130
30	226.000	0.099	0.035	232.030	22.660	18.390	392.520	78.080	4.246	31.200	1.400
31	173.780	0.023	0.070	187.020	52.990	20.175	428.400	80.447	3.987	20.880	0.950
32	192.410	0.002	0.079	195.940	49.230	19.380	459.000	63.467	3.275	24.240	1.280
33	366.420	0.060	0.017	376.820	50.250	19.185	320.080	48.653	2.536	36.060	0.990
34	300.820	0.052	0.043	305.100	36.870	12.765	268.790	56.947	4.461	23.200	1.510
35	175.360	0.033	0.023	186.840	28.980	12.000	377.160	49.553	4.129	24.360	1.010
36	269.060	0.106	0.014	279.810	20.920	17.700	324.360	135.907	7.678	23.280	0.870
37	334.490	0.023	0.042	343.630	33.250	21.960	384.360	66.280	3.018	30.060	1.250
38	324.220	0.008	0.050	332.400	12.230	14.445	387.480	28.360	1.963	31.680	0.943
39	259.230	0.020	0.014	270.730	15.160	25.215	393.000	92.227	3.658	24.960	1.080
40	231.190	0.019	0.018	239.120	26.300	26.265	433.080	111.487	4.245	27.240	1.190
41	329.550	0.056	0.073	337.100	12.860	21.270	248.040	41.720	1.961	39.770	1.330
42	365.280	0.013	0.021	371.160	26.370	21.960	466.080	112.113	5.105	29.520	1.750
43	392.430	0.038	0.011	403.440	15.520	15.330	480.480	88.247	5.756	29.040	1.570
44	299.520	0.056	0.022	303.880	24.710	13.050	523.200	71.680	5.493	25.200	1.250
45	251.340	0.012	0.009	257.430	13.910	12.060	342.360	72.673	6.026	28.680	1.020
46	265.660	0.021	0.007	275.620	26.830	16.040	443.160	113.013	7.046	25.440	0.880
47	306.950	0.046	0.014	310.930	19.170	22.455	291.600	96.080	4.279	25.440	0.960
48	184.830	0.009	0.019	190.990	27.700	19.050	350.520	118.800	6.236	28.800	0.790
49	365.700	0.012	0.010	373.000	24.600	18.690	259.330	110.400	5.907	32.880	1.110
50	268.860	0.043	0.002	276.910	27.780	23.235	269.520	87.920	3.784	36.120	1.240
51	364.330	0.050	0.014	373.540	26.790	14.040	372.720	129.280	9.208	24.360	1.180
52	222.440	0.047	0.008	227.440	37.200	19.312	255.480	114.193	5.913	30.360	0.910
53	330.420	0.034	0.013	339.060	32.190	16.480	269.040	73.047	4.432	18.650	0.840
54	386.460	0.004	0.013	392.470	25.190	20.637	266.436	105.107	5.093	28.800	0.740
55	310.530	0.027	0.008	315.330	29.620	18.513	289.680	21.013	1.135	21.600	0.880
56	269.210	0.037	0.034	273.810	30.090	14.195	258.720	93.553	6.591	29.160	0.960
57	278.840	0.007	0.012	285.670	26.920	18.440	248.160	101.440	5.501	23.280	1.150
58	257.460	0.008	0.012	262.730	29.590	19.529	397.760	123.413	6.319	25.920	1.220
59	336.110	0.036	0.009	341.470	29.720	18.368	377.120	85.600	4.660	16.800	0.670
60	320.560	0.026	0.021	328.030	37.520	18.077	517.120	129.093	7.141	27.820	1.320
61	288.170	0.049	0.015	296.420	33.450	19.310	542.880	87.887	4.551	27.840	0.943
62	224.710	0.031	0.023	230.790	27.210	15.173	332.150	92.240	6.079	30.360	1.130
63	305.590	0.047	0.010	313.500	30.230	11.616	322.720	53.833	4.634	18.840	1.740
64	293.210	0.031	0.044	299.000	34.570	19.965	264.480	112.607	5.640	25.320	0.770
65	169.690	0.009	0.032	171.770	29.030	14.956	451.100	78.720	5.264	17.880	0.920
66	325.600	0.015	0.018	335.970	42.800	24.600	375.760	147.733	6.005	18.120	0.780
67	132.470	0.013	0.033	134.940	33.580	16.117	348.640	35.240	2.186	32.150	1.030
68	406.440	0.034	0.022	415.430	38.820	15.972	296.960	134.713	8.434	18.240	1.120
69	238.230	0.019	0.017	241.420	18.250	16.214	554.240	105.767	6.523	28.800	0.720
70	221.980	0.064	0.018	231.190	28.850	20.570	300.320	71.547	3.478	15.360	0.690
71	296.400	0.033	0.016	305.390	30.620	18.513	285.040	78.973	4.266	22.320	0.838
72	332.710	0.045	0.018	344.960	35.040	14.593	470.480	124.513	8.533	18.240	0.960
73	237.210	0.014	0.018	243.740	28.940	25.555	302.480	47.973	1.877	22.440	1.130
74	280.290	0.030	0.017	284.440	27.500	17.206	431.120	51.273	2.980	25.940	1.400
75	339.930	0.029	0.015	342.030	33.150	14.665	569.520	69.753	4.756	37.470	0.950
76	275.850	0.027	0.017	279.100	22.920	22.288	361.040	84.073	3.772	34.780	1.280
77	207.450	0.039	0.029	210.190	24.140	16.625	476.000	103.087	6.201	25.860	0.990
78	307.380	0.018	0.017	310.170	27.890	20.618	486.160	139.287	6.755	20.910	1.510
79	282.640	0.099	0.018	284.580	21.440	17.061	502.880	78.847	4.621	29.900	1.010
80	254.640	0.077	0.028	261.320	27.040	16.408	449.600	90.733	5.530	20.450	0.870
81	258.160	0.072	0.065	261.230	13.060	24.330	175.800	92.253	3.792	18.400	1.250
82	268.260	0.125	0.017	271.150	23.180	19.965	203.272	126.453	6.334	29.360	1.130
83	288.760	0.103	0.018	295.730	29.440	16.916	368.264	87.133	5.151	29.900	1.080
84	252.790	0.111	0.016	262.140	20.760	25.483	259.696	152.673	5.991	21.610	1.190
85	301.240	0.093	0.030	304.950	45.640	18.005	408.936	103.440	5.745	28.790	1.330
86	157.320	0.133	0.015	162.600	58.850	21.199	484.856	131.200	6.189	29.800	1.760
87	276.470	0.151	0.019	283.180	34.300	13.504	555.420	79.493	5.887	28.680	1.570
88	306.620	0.088	0.013	312.740	26.340	24.810	429.504	113.920	4.592	17.070	0.792
89	291.870	0.101	0.019	296.180	33.870	20.860	333.040	109.547	5.252	27.170	1.220
90	280.660	0.122	0.016	285.210	22.870	14.084	164.376	55.707	3.955	20.100	0.951
91	226.940	0.145	0.026	229.790	29.170	9.365	241.380	34.427	3.676	26.560	1.320
92	251.010	0.078	0.016	257.750	47.260	18.440	237.348	93.093	5.048	34.040	0.702
93	258.020	0.161	0.011	261.910	19.650	12.995	353.808	106.000	8.157	29.800	1.420
94	291.100	0.088	0.011	298.460	33.680	16.117	374.440	85.600	5.311	30.010	0.738
95	257.360	0.145	0.016	261.560	33.320	10.600	234.912	75.840	7.155	29.800	0.936
96	212.940	0.134	0.021	219.140	29.780	18.513	522.000	86.600	4.678	36.870	0.680
97	241.690	0.151	0.012	249.950	16.280	19.275	188.916	107.320	5.568	23.740	0.890
98	265.180	0.148	0.023	269.290	31.490	20.038	274.788	74.180	3.702	28.790	1.200
99	294.090	0.122	0.012	304.470	27.140	20.400	276.852	49.920	2.447	17.070	0.845
100	263.890	0.095	0.025	266.680	22.460	17.680	213.096	49.120	2.778	20.970	1.150
101	250.810	0.144	0.010	256.720	30.210	20.320	284.124	66.453	3.270	26.520	1.180
102	208.160	0.156	0.008	213.240	16.250	21.520	365.544	103.100	4.791	19.800	1.240
103	321.010	0.123	0.016	327.770	22.280	20.240	325.344	51.887	2.564	20.490	0.900
104	268.970	0.137	0.021	274.690	24.510	19.920	215.916	137.320	6.894	30.100	1.190
105	351.210	0.110	0.020	353.540	18.910	18.890	291.372	108.880	5.764	26.660	1.200
106	276.170	0.102	0.011	280.300	24.025	16.720	388.596	117.840	7.048	34.140	0.900
107	293.460	0.110	0.034	305.690	22.837	12.800	280.704	28.880	2.256	36.870	0.890
108	242.490	0.115	0.073	250.700	27.240	22.000	314.916	28.720	1.305	34.380	1.200
109	333.730	0.099	0.077	338.930	15.660	16.480	468.716	33.280	2.019	18.690	1.230
110	300.480	0.077	0.136	307.120	30.747	16.080	324.120	63.440	3.945	14.340	0.834
111	260.010	0.072	0.055	262.960	24.287	17.760	245.292	52.320	2.946	26.160	1.400
112	245.770	0.125	0.048	249.920	28.220	17.600	287.840	31.840	1.809	19.090	1.320
113	236.850	0.103	0.059	241.260	17.153	17.867	582.840	74.240	4.155	18.990	0.756
114	276.370	0.111	0.069	286.070	40.877	22.667	433.800	56.480	2.492	22.560	1.330
115	148.980	0.093	0.097	152.110	44.297	20.400	508.080	52.240	2.561	27.370	0.859
116	282.570	0.133	0.030	286.650	38.190	16.080	258.360	50.480	3.139	25.650	0.880
117	214.960	0.151	0.105	219.370	38.160	28.160	280.520	52.000	1.847	29.900	0.750
118	268.510	0.088	0.038	274.150	26.400	18.960	388.752	122.960	6.485	15.550	1.330
119	230.490	0.101	0.099	236.340	32.640	16.160	367.404	93.280	5.772	22.030	1.540
120	354.970	0.122	0.035	358.920	30.743	24.560	483.972	88.800	3.616	24.140	0.980
121	297.900	0.145	0.040	301.880	26.883	18.320	350.556	86.400	4.716	20.940	0.790
122	337.150	0.078	0.064	343.300	40.633	22.720	407.992	40.160	1.768	25.490	0.584
123	338.720	0.161	0.075	342.680	28.846	18.800	590.880	73.680	3.919	19.720	0.683
124	200.200	0.088	0.056	210.460	29.018	18.080	524.988	104.480	5.779	16.270	0.440
125	298.480	0.145	0.051	303.810	33.291	12.480	292.212	40.000	3.205	19.220	0.735
126	249.990	0.134	0.064	258.220	27.460	22.000	466.932	42.960	1.953	30.780	0.741
127	236.140	0.151	0.091	242.220	40.407	18.640	332.460	74.560	4.000	30.640	0.987
128	263.560	0.148	0.091	268.430	26.554	28.080	353.592	64.240	2.288	33.610	1.510
129	198.620	0.122	0.088	205.580	27.537	19.840	265.284	59.840	3.016	19.800	0.673
130	192.190	0.095	0.083	198.190	44.201	23.360	249.356	111.120	4.757	38.220	1.070
131	274.230	0.144	0.042	278.730	45.123	14.880	343.680	80.240	5.392	30.840	1.580
132	238.230	0.156	0.065	244.930	38.445	16.320	347.520	64.240	3.936	23.640	1.170
133	222.620	0.123	0.022	229.190	23.670	15.680	323.400	49.920	3.184	22.310	1.080
134	187.450	0.137	0.019	192.510	25.465	15.520	458.172	102.480	6.603	24.310	0.650
135	179.990	0.110	0.021	187.010	42.510	10.320	424.992	60.800	5.891	27.600	0.940
136	156.780	0.102	0.052	161.760	15.545	20.320	269.120	67.920	3.343	20.160	0.810
137	200.460	0.110	0.095	206.140	40.525	14.320	295.480	82.160	5.737	18.840	1.130
138	346.870	0.115	0.052	352.400	21.240	17.760	439.044	39.200	2.207	32.560	1.080
139	327.110	0.060	0.040	333.540	25.400	11.680	348.448	79.600	6.815	19.800	0.620
140	213.770	0.007	0.094	219.180	21.820	20.400	450.060	35.680	1.749	21.720	0.950
141	234.680	0.060	0.056	240.890	22.680	21.240	183.648	45.360	2.136	23.640	0.917
142	186.450	0.015	0.086	193.370	25.205	22.080	467.484	45.360	2.054	20.760	1.017
143	119.890	0.024	0.085	126.560	21.680	17.200	417.028	51.520	2.995	18.960	1.110
144	178.320	0.007	0.059	184.900	34.190	17.760	433.360	45.200	2.545	16.080	1.042
145	112.330	0.016	0.063	118.090	23.295	15.400	390.552	101.760	6.608	31.440	0.776
146	146.540	0.121	0.035	152.450	23.400	20.560	446.832	136.000	6.615	28.960	1.083
147	222.580	0.022	0.050	229.280	33.684	9.760	237.936	68.533	7.022	18.400	0.842
148	234.330	0.031	0.098	240.840	40.387	11.360	369.196	59.280	5.218	16.640	0.983
149	236.840	0.063	0.053	241.940	21.110	18.160	306.504	140.000	7.709	25.600	0.633
150	267.000	0.106	0.041	271.610	41.237	10.560	316.044	27.200	2.576	24.320	1.058
151	286.740	0.028	0.032	292.010	35.663	15.120	440.280	39.680	2.624	23.520	1.067
152	321.180	0.017	0.037	325.310	52.482	18.800	196.640	68.160	3.626	17.280	1.083
153	307.830	0.123	0.050	315.060	58.310	11.360	412.800	58.800	5.176	21.840	0.925
154	298.450	0.069	0.041	303.080	57.830	29.280	373.560	57.200	1.954	29.200	0.942
155	338.220	0.028	0.046	344.650	30.860	17.040	490.680	77.200	4.531	26.070	0.942
156	255.850	0.014	0.042	259.690	62.542	24.960	263.080	129.200	5.176	32.590	0.958
157	265.170	0.141	0.086	269.450	34.066	27.360	506.400	112.240	4.102	20.720	0.958
158	241.030	0.126	0.081	245.150	21.740	26.040	464.760	101.200	3.886	22.960	0.958
159	323.640	0.028	0.052	329.120	41.601	24.120	339.800	130.080	5.393	28.080	0.701
160	268.090	0.023	0.056	273.950	42.610	21.360	292.200	110.133	5.156	24.440	0.819
161	309.950	0.041	0.046	316.960	31.460	28.080	372.240	77.707	2.767	26.670	0.528
162	281.840	0.027	0.011	288.020	28.880	16.200	524.400	103.120	6.365	27.780	0.882
163	299.060	0.072	0.021	304.060	40.170	28.440	496.080	78.080	2.745	17.310	0.889
Mean	270.455	0.066	0.037	276.795	30.903	18.820	364.392	81.535	4.470	25.030	1.051
S.D.	57.901	0.048	0.026	58.450	9.656	4.220	95.502	31.485	1.779	5.780	0.276
